# Explaining Children’s Life Outcomes: Parental Socioeconomic Status, Intelligence and Neurocognitive Factors in a Dynamic Life Cycle Model

**DOI:** 10.1007/s12187-017-9481-8

**Published:** 2017-06-26

**Authors:** Elise de Neubourg, Lex Borghans, Karien Coppens, Maria Jansen

**Affiliations:** 1 School of Business and Economics, Maastricht University, Tongersestraat 53, 6211 LM, Maastricht, The Netherlands; 2 School for Public Health and Primary Care, Maastricht University, Maastricht, The Netherlands

**Keywords:** Child development, Inequality, Life cycle, Executive functions, Socioeconomic status, Non-cognitive abilities

## Abstract

The goal of the present paper is to introduce a dynamic life cycle model that explains the reproduction of wealth and health over generations by introducing knowledge from cognitive neuroscience into the existing knowledge from the socioeconomic literature. The socioeconomic literature successfully identified the major role of socioeconomic status of parents, both as a direct and indirect effect, in the shaping and intergenerational reproduction of wealth and health. Furthermore, the importance of cognitive abilities as actor in this process has been widely studied in socioeconomic literature. A third factor that has been identified by the socioeconomic discipline is the so-called non-cognitive capabilities. This category, however, lacks a clear definition and seems to be a catchall for a collection of factors. Within the repository of these non-cognitive capabilities the construct of executive functions is an interesting and important contribution from cognitive neuroscience. The multidimensional construct of executive functioning or cognitive control (i.e. planning and formulation of objectives) and monitoring processes (i.e. influences the execution of these goals) is a valuable addition to a framework on reproduction of wealth and health over generations, because executive functions are sensitive to training. Merging insights of the socioeconomic literature and cognitive neuroscience in a life cycle model opens the opportunity of educational initiatives with regard to executive functions to break the intergenerational reproduction of poverty and deprivation.

## Introduction

Despite notable advances during the last century in reducing economic inequalities at large, the result is still described in terms of a big gap between the wealth and health of the top compared to the wealth and health of the majority in the middle- and at the bottom of the distribution. More importantly, the gap between the very bottom and the rest of the society is still big, and (intergenerational) upward mobility is limited, in other words children from families with a low socioeconomic status are very likely to stay in that very bottom part of the distribution throughout their life (UNICEF 2013). Given the inequality problem as described above, finding ways to improve upward mobility is of major relevance.

The lack of upward mobility from the bottom can be related to three main categories of differences between the poor and the wealthier; differences in socioeconomic options and possibilities, differences in cognitive abilities, and differences in non-cognitive capacities (Bradley and Corwyn 2002; Heckman et al. 2006). The underlying realities for the first two categories have proven to be quite stable over a lifetime and seem therefore unsuitable to play a role in breaking the cycle of reproduction of wealth and health from one generation to the next one (Devlin et al. 2013; Altzinger et al. 2015). The third category, non-cognitive abilities, lacks a clear definition and seems to be a catchall term for individual factors other than intelligence. Yet, the specification in cognitive neuroscience literature of the concept of executive functioning can be used to (re)define non-cognitive abilities (Belfi et al. 2015). Merging the knowledge from cognitive neuroscience into the existing socioeconomic frameworks on intergenerational reproduction of wealth and health into a dynamic life cycle model on the reproduction of wealth and health together with exploring the trainability of executive functioning could potentially reveal a method to break the cycle of poverty. Hereafter, we discuss the three categories explaining the differences between the poor and the wealthy in more detail and we elaborate on how these factors could be merged to create a single dynamic life cycle model on the reproduction of wealth and health.

Socioeconomic status as measured by the highest level of education of a child’s parents, the level of income, and/or the status of the occupation of a child’s parents is said to play a decisive role in maintaining inequality; adults who, as children, have been nurtured in (relatively) higher-wealth families experience better school results, higher earnings and healthier- and longer healthy lives than adults who grew up in less wealthy families (Altzinger et al. 2015). The studies on the reproduction of inequality provide a gloomy perspective about the fate of the most unprivileged in our societies: when born in an underprivileged family one’s chances to do much better than his/her parents, are small.

A second category of differences between the poor and the wealthier accounting for the lack of upward mobility from the bottom is related to cognitive abilities as measured by IQ. Individual differences in intelligence are largely due to genetic variation (Devlin et al. 1997; Davies et al. 2011; Trzaskowski et al. 2014). There is an on-going discussion about the degree by which levels of intelligence could be changed by investments (e.g. education). The current state of the debate seems to conclude that changes are marginal at best (Devlin et al. 2013). However, even if intelligence is mainly genetically coded, it is plausible that someone’s level of intelligence might be fixed but that certain external circumstances may result in a suboptimal use of the pre-set intelligence. It is also possible that IQ in interaction with other (non-cognitive) factors may lead to better life outcomes then IQ alone would predict.^1^

The third category of differences between the poor and the wealthier, and increasingly considered relevant for explaining the (intergenerational) reproduction of inequality is related to so-called non-cognitive abilities. These “non-cognitive abilities”, however, lack a clear definition although concepts like “personal preferences”, “thinking styles” and “personality traits” are often used to capture these non-cognitive abilities (Heckman et al. 2006) . Exactly within the repository of these non-cognitive abilities, there is an important contribution from the neurocognitive scientific literature. In this literature “non-cognitive abilities” are specified as “executive functioning” or “cognitive control”. This multidimensional construct influences cognitive skills during planning and the formulation of objectives on the one hand and effectively influences the execution of these plans on the other (Diamond 2013).

Studies in neuroscience consistently find that children’s performance on executive functions-tasks is a very good predictor of wellbeing and welfare later in life: children showing better performance in the dimensions of executive functioning (inhibitory control, working memory and cognitive flexibility) grow into healthier, wealthier and better educated adults leading more balanced and more stable lives (Diamond 2013). This predictive value of executive functioning cannot be viewed isolated because the development of executive functions within children is influenced by external circumstances during pregnancy, early childhood, primary school-age and adolescence, including parents’ behaviour, peer pressure and education (Lee et al. 2013).

This paper explores the possibilities for upward mobility through development of executive functioning, while reconciling these three findings (i.e. the influence of socioeconomic status, cognitive abilities and non-cognitive abilities on the development of wealth and health) and their underlying theories. It does this by formulating a framework in which the mechanisms of the development of executive functioning and cognitive abilities, the place of the parental household in the wealth distribution of a given society and the quality of the external circumstances are linked. The full formulation of this theoretical framework needs to go well beyond the intuitive direct links between the socioeconomic status of households and the development of executive functions. The introduced theoretical framework attempts to solve part of the puzzle of intergenerational reproduction of wealth and health by reorganizing the relevant factors into five big categories (health, executive functions, cognitive development, external circumstances and direct and indirect influence of parents) in an integrated dynamic framework that explains the drivers of the reproduction of wealth and health over generations.

To summarize, economic studies on the reproduction of inequality tell a disappointing story about the fate of the most unprivileged in our societies. However, understanding one of the (major) mechanisms on the neurocognitive development (i.e. development of executive functioning), investigating its responsibility for the reproduction of inequality and its dynamic relationships with the other drivers of inequality may provide a more optimistic view. Executive functions, can be trained. This means that if executive functions play an observable role in yielding economic and social success and if early childhood initiatives and other forms of education can contribute to the fuller development of executive functions, then the latter educational initiatives could contribute significantly to breaking the intergenerational reproduction of poverty and deprivation.

The purpose of this paper is to introduce a dynamic life cycle model explaining the reproduction of wealth and health by merging the perspectives of two sets of disciplinary insights. Given the comprehensive existing literature surveys summarizing the influence of the socioeconomic status of parents on the wealth and health of their children (Sirin 2005; Haveman and Wolfe 1995; Altzinger et al. 2015) we will only briefly recapitulate these, the paper focuses on the newer insights of neuropsychological studies (section 3) and the integration of them in a dynamic life cycle model (section 4). Finally the relevance of the dynamic life cycle framework in terms of policy and empirical questions is discussed in the concluding section.

## The Reproduction of Wealth and Health: The Socioeconomic Perspective

The socioeconomic literature on the intergenerational transmission of wealth and health, distinguishes the three main drivers of inequality: socioeconomic status, cognitive abilities, and non-cognitive abilities (Cunha and Heckman 2009).

### Socioeconomic Status

As Altzinger et al. conclude (2015): “Parents have a strong influence on their descendants outcomes.” The construct of socioeconomic status is probably one of the most widely used contextual variables in the socioeconomic literature and usually refers to three main indicators: parental income, parental level of education, and parental occupation (Sirin 2005). Parental income is seen as a reflection of the available resources in the household for the developing child both economically and socially(Duncan et al. 2007) . The level of parental education is thought to influence the educational attainment of their offspring. Children of highly educated parents tend achieve a higher level education, this is due to the direct effect of having (genetically) a higher ability and to the indirect effect of having more educated parents as a role model (Black and Devereux 2011). Parental education has been reported to be the most stable indicator of socioeconomic status because it is mostly established early in life and remains the same over time (Sirin 2005). The third indicator -parental occupation- gives information about the prestige and culture attached to an occupation and is being built upon the level of education and income related to the occupation (Sirin 2005).

The socioeconomic status of parents is thought to have both a direct and an indirect effect on their children. The direct effect is described by the model of Leibowitz (1974) and the utility function approach of Becker and Tomes (1979); the level of family income, which is under the influence of parents abilities and level of education, determines the home investments, which in turn effects children’s final schooling level leading to children’s earnings and income. Home investments are determined by the quality and quantity of both time and goods inputs parents invest in their children (Leibowitz 1974). This leads to the conclusion that parents with greater access to economic resources are more able to invest in their offspring’s education (Haveman and Wolfe 1995). Furthermore, socioeconomic inequalities between families play a role in the determination health; the healthy life expectancy for people coming from lowest socioeconomic background typically is ten years less compared to those coming from high socioeconomic backgrounds (Mackenbach 2015).^2^

The indirect effect of parental socioeconomic status on children can be described by means of ecological theories for example see Dahlgren and Whitehead (1991) and Bronfenbrenner (1986). These ecological theories attempt to map the relationship between the individual, their direct environment and society, in one model. In the middle of the model stands the developing child, and the environment is divided into four layers surrounding the child (Bronfenbrenner 1986). Each layer has a different influence on the child. The inner layer is the so-called “micro system”, this is the closest to the child, and includes among others, family, school and neighbourhood. This layer is characterized by the reciprocal influences of the factors within. The next layer the “meso-system”, represents the connections between the actors in the micro system, such as the relationship between parent and teacher. The third layer is the “exo-system”. The child is not a direct part of this layer but parents are. The main example of this layer is the work environment of parents. The outer layer is the “macro-system” which represents the society in which the child lives, including public investments, laws and values.

According to these ecological models, the socioeconomic status of parents will have a direct effect on the development of a child through the micro system but also an indirect effect through the outer layers of the model, thus making it a strong predictor of life success of a child.

Table 1 provides a summary of some of the main studies on the effect of the socioeconomic status on a number of life outcomes. These are selected from a long list of life outcomes affected by socioeconomic status. They reflect the focus in this article and in strengthen the theoretical framework introduced in section 4.

**Table 1:** Summary of recent studies on socioeconomic status as a relevant factor for life outcomes

Aspect of life	In which way is socioeconomic status relevant?	References
Physical health	- Individuals who are observed to have a better health status tend to be better educated, regardless how health is measured - In all high income countries with available data the health expectancy between the highest and the lowest socioeconomic groups typically differs 10 years or more - The relationship between household income and a child’s health becomes more clear as children grow older	Mackenbach (2015) Auld and Sidhu (2005) Case et al. (2002)
School readiness	- A child coming from a low-income family has a twice as big of change of being vulnerable for school readiness	Janus and Duku (2007)
School success	- Poverty and low parental education are associated with lower levels of school achievement and high levels of high school drop-out rates	Bradley and Corwyn (2002)
Job success	- Son’s earnings correlate highly with fathers earnings - Occupational rank or prestige of parents correlates with children’s occupation	Black and Devereux (2011) Solon (2002)

One general outcome that studies in this area share, is that children growing up in lower social-economic status families are worse off, whether it is measured in educational attainment, labour participation or income compared to those children growing up in higher socioeconomic status families.

### Cognitive Abilities

Socioeconomic literature also takes two categories of psychological influences into account when talking about life outcomes (i.e. those mentioned in Table 1). These influences can roughly be dived into two categories: cognitive and so-called non-cognitive abilities.

In socioeconomic research “cognitive abilities” are usually measured by *g,* which stands for general intelligence or IQ (Gottfredson 2002). Numerous studies have established that cognitive ability measured by general intelligence tests is a predictor of schooling attainment and wages (Cawley et al. 2001; Gottfredson 2002; Heckman et al. 2006). But to what extent this finding is relevant for social policy has been widely discussed over the last decades. There is an agreement that the base of IQ is largely defined by DNA (Devlin et al. 1997; Trzaskowski et al. 2014; Davies et al. 2011) but the level of changeability and therefore the possible level influence of educational training on cognitive abilities is still unclear (see for example, Cawley et al. (2001), Devlin et al. (1997)).

Cognitive abilities are not seen as isolated phenomena. For example Cunha et al. (2006) finds that the formation of cognitive skills is a life cycle process where, besides the large genetically marker, parents and families play an important role in the development of the cognitive skills of their children. Mani et al. (2013) have studied the direct effect of poverty on cognitive abilities among adults. Their analysis focuses on the short term ‘cognitive overload’ effect of poverty and their findings strongly demonstrate that poor people have more difficulties in performing well then non-poor: however the direct effects of poverty can no longer be observed, among the same people when no longer poor.

### Non-cognitive Abilities

The second kind of psychological influences taken into account in socioeconomic studies are the so-called non-cognitive abilities. Non-cognitive abilities have been described in the socioeconomic literature as “personality traits, persistence and motivation” (Heckman et al. 2006). They are believed to be important because in the right combination they may enhance the cognitive abilities of people. Duckworth and Gross (2014) find that the ability to resist temptation and the ability to pursue a dominant goal, also referred to by the concept of “grit”, are related but distinct, both abilities need to be combined to result in successful behaviour.

In the mid-60ies, the “Perry preschool program” was introduced in the USA. The program targeted African American children coming from a low socioeconomic background. The goal was to enhance the cognitive skills of the participants from age 3 to 5 years of age. Immediately after the program the first results showed an improvement in cognitive abilities but this effect faded a few years after the program was finished. The absence of a long-term IQ effect was seen a failure of the program (Heckman et al. 2013). However, when the participants reached adulthood, a longitudinal study showed significant positive outcomes not only in the level of education reached but also in other domains such as employment, earnings, marriage and health (Heckman et al. 2013). This led the researchers to conclude that there is another group of abilities besides cognitive abilities that is active in shaping life outcomes (Heckman et al. 2013). This group of abilities is captured by the catchall concept “non-cognitive” skills.

### Implications for Life Cycle Model

To summarize, controlling for cognitive abilities, the parental socioeconomic status can be seen as a good proxy for the combined influence of the investments made in children, the harm done to children, the quality of their environment and the opportunities for development they encounter.

While that is interesting from an academic point of view, it leads to a pessimistic view regarding intergenerational mobility because the socioeconomic status of parents is difficult to change. Moreover changes in status may trickle down too slowly to make a significant change in the life of children.^3^ Literature indicates that non-cognitive abilities shows great potential as a mechanism to effectively stimulate upwards mobility and make a positive change in children’s lives.

Non-cognitive abilities are often introduced as a catchall concept for all individual factors other than intelligence However, by using recent knowledge from the neurocognitive literature concerning executive functions; a clearer definition of the concept of “non-cognitive abilities” can be given. The next section of this paper will further elaborate on the definition, predictive value and development of executive functions.

## Executive Functions: Definition and Predictor for Life Outcomes

Executive functions are a complex multidimensional interrelated construct of cognitive control- and monitoring processes, influencing cognitive skills regulating to formulation of goals, planning how to achieve them, and carrying out these plans effectively. They are also referred to as cognitive control.

There is a general agreement that there are three core executive functions: inhibition, working memory, and cognitive flexibility (Miyake et al. 2000) A substantial amount of research on executive functions is on the unity /diversity framework. According to this framework the three core executive functions can be measured separable (diversity) but they are correlated and have a common underlying ability (unity) (Miyake and Friedman 2012). Emerging from the three executive functions are problem solving and reasoning, which are defined as higher order executive functions (Diamond 2013). We use this unit/diversity framework to study individual differences in executive functions.

Below, we describe the relevance of each of these three core executive functions for child development. Each function is defined and discussed in terms of its measurement, its relevance for every day functioning, and its relevance for the theoretical framework of our research.

### Inhibition

Inhibition or inhibitory control refers to the conscious and unconscious monitoring of attention, behaviour, thoughts and emotions by supressing distracting stimuli or a strong internal predisposition (Diamond 2013). Inhibition plays an important role in everyday life and especially in classroom settings. In order to assess inhibitory control the literature describes three kinds of psychological tasks. The first measures to which extend a person is able to delay gratification in order to benefit from it. The ‘marshmallow test’, for example, involves placing one snack in front of young children (3 to 5 years of age) and asking them to wait before taking it. If they postpone the eating of the snack the children can have more than one snack as a reward (Mischel 2014).

A second way to measure inhibition control is to ask participants to respond to a certain stimulus and ignore the surrounding stimuli. For example, through the so-called Stroop task, Simon task and Flanker task. The Stroop task measures to which extend children are able to ignore meaning and to focus on superficial characteristics such as font, style or colour of the words. During the test participants are asked to ignore the meaning of the word (i.e. inhibit the dominant response to words) and instead report the colour in which the word is presented. Another type of measurements of inhibitory control is the go/no-go and stop-signal tasks. During these tasks a participant is usually asked to respond by pressing a button if a specified stimulus appears, but not to react when another specific stimulus appears. These tasks don’t ask participants to inhibit one response to make another; they simply inhibit a response to do nothing (Diamond 2013).

### Working Memory

Working memory involves holding information in mind and mentally working with it (Baddeley and Hitch 1994). Working memory is not to be confused with short-term memory. The difference is that working memory refers to not only remembering the information but also being able to work with information when it is no longer visible. The relevance of working memory capacity is that it is necessary for a large number of everyday actions like translating instructions into action plans, incorporating new information into one’s thinking or action plans (updating), considering alternatives, and mentally relating information to formulate a general principle (Diamond 2013). Working memory is measured with the use of tasks that require a person to remember two rules at the same time. For example subjects are confronted with alternating series of stimulus 1 and stimulus 2. They are given the instruction to press on the same side as the stimulus when shown stimulus 1 and press on the side opposite to the stimulus when shown stimulus 2. This task requires the subject to not just hold the two rules in mind but also to be mentally translate the rules on the present stimulus, and therefore measures the effectiveness of a person’s working memory.

### Cognitive Flexibility

Cognitive flexibility is also referred to as “shifting”. Cognitive flexibility is needed when shifting back and forth between multiple tasks or mental operations (Miyake et al. 2000). To make a “shift” we need to inhibit our previous perspective and load into our working memory a new perspective. Therefore, cognitive flexibility is related to the two previously described functions as it builds on inhibitory control and working memory (Diamond 2013). In education, the aim is to help students to learn as well as to appropriately apply and adapt what they have learned to novel situations. For the latter, cognitive flexibility is crucial. Tasks that are regularly used for measuring cognitive flexibility are so called “task-switching tasks” where a person is asked to perform two separate tasks. In the third condition the two tasks are mixed and the reaction time between the tasks is seen as the switching cost. This test is used as a measurement of cognitive flexibility.

### Executive Functions versus IQ

At first sight the construct of executive functioning shows similarities to the construct of intelligence (IQ). Neuroscientific research has shown that executive functioning is highly correlated with frontal lobe brain activity. Individuals with frontal lobe damage, and more specifically prefrontal cortex damage, show deficits in executive functioning but appear to have normal intelligence. For example, Friedman et al. (2006) studied the relation between measurements of the three executive functions and intelligence by correlating the results of Wechsler Adult Intelligence Scale (WAIS) with tasks measuring the three executive functions. Inhibition and cognitive flexibility showed no or little correlation with the results of the WIAS. The intelligence measurement did share a significant amount of variances with working memory performance. Ackerman et al. (2005) studied the relation between working memory performance and general intelligence measurements in more depth. They concluded that, despite similarities, they are not the same constructs and are not interchangeable.

### Development of Executive Functions

An essential question for the in this paper presented lifecycle model is: which factors influence the development of children’s executive functions? From the literature, we know that several factors influence the structure and functioning of the prefrontal cortex, and consequently, that affects someone’s executive functioning. These factors can roughly be divided into two categories: factors that influence early (prenatal) development of the prefrontal cortex and factors that influence the effectiveness of the prefrontal cortex.

The first category is a group of factors that have an inception before the child is born. As described earlier, executive functions rely on the functioning of the frontal lobe, more specifically the prefrontal cortex. The prefrontal cortex is a very sensitive region of the human brain during development that starts to develop already during pregnancy. Prenatal exposure to substances such as alcohol, nicotine and cannabis have been related to adverse neurobehavioral and cognitive outcomes (Huizink and Mulder 2006). These effects of maternal substance use during pregnancy were measured in children beyond the age of 3 years old. Findings suggest that executive functioning is negatively associated with prenatal substance abuse. Prenatal substance abuse leads to a higher probability for the development of behavioural and learning problems (Huizink and Mulder 2006).

Over the last decades, neonatal care has significantly reduced the mortality rate of preterm infants. However, preterm children often face challenges in behavioural control, social-emotional ability, and school performance later in life. These challenges could be caused by deficits in the development of the prefrontal cortex. Ni et al. (2011) investigated whether 6 years old children born as preterm infants with very low birth weight but with normal early development still have executive functioning deficits. They concluded that even with “normal early development” at the age of 6 years old, children born with very low birth weight are still at risk of deficits in planning, cognitive flexibility, and nonverbal working memory (Ni et al. 2011).

The second category of factors influencing executive functioning is related to life events and conditions during childhood. Executive functioning and prefrontal cortex are still vulnerable to some external factors after birth. Executive functions are majorly affected, if a person is stressed, sad, lonely, sleep deprived, or not physically fit (Diamond 2013). Liston et al. (2009) used functional magnetic resonance imaging (fMRI) to investigate the effect of chronic stress on the functioning of the prefrontal cortex while executing an attention-shifting task. Due to the stress, control of attention was selectively impaired and functional connectivity within a frontopariental network that mediates attention shifts was disrupted. However, the found effects were reversible; after a month of reduced stress, the same subjects showed no significant differences from control (Liston et al. 2009).

### Influence of Executive Functions on Life Outcomes

Executive functions are an essential input variable when building a theoretical framework concerning child development because they have a high predictive value for numerous life outcomes: “Purely cognitive measures of executive functions can predict individual differences in clinically and societally important behaviours” (Miyake and Friedman 2012). The relationship between executive functioning and educational attainment is especially pronounced. Several recent studies relate executive functioning early in life to school readiness, academic achievement, health outcomes, and success later in life (see Table 2, based on (Diamond 2013)). As both Tables 1 and 2 make clear, executive functions and socioeconomic status both play an important role in the same life outcomes. This suggests that the effect of executive functioning on life outcomes cannot be viewed isolated from the effects of the socioeconomic context in which a child is grows up. Therefore dynamic life cycle model introduced incorporates both the effect of socioeconomic context and the effect of executive functioning of life outcomes.

**Table 2:** Summary of recent studies on executive functions as a predictor for life outcomes. Source: (Diamond 2013)

Aspects of life	In which way are executive functions relevant?	References
Physical health	Poorer EFs are associated with obesity, overeating, substance abuse, and poor treatment adherence	Crescioni et al. (2011), Miller et al. (2011), Riggs et al. (2010)
School readiness	EFs are more important predictors for school readiness than are IQ or entry-level reading or math	Blair and Razza (2007), Morrison et al. (2010)
School success	EFs predict both math and reading competence throughout the school years	Borella et al. (2010), Duncan et al. (2007), Gathercole et al. (2004)
Job success	Poor EFs lead to poor productivity and difficulty finding and keeping a job	Bailey (2007)

## Dynamic Life Cycle Model

**Fig. 1:**
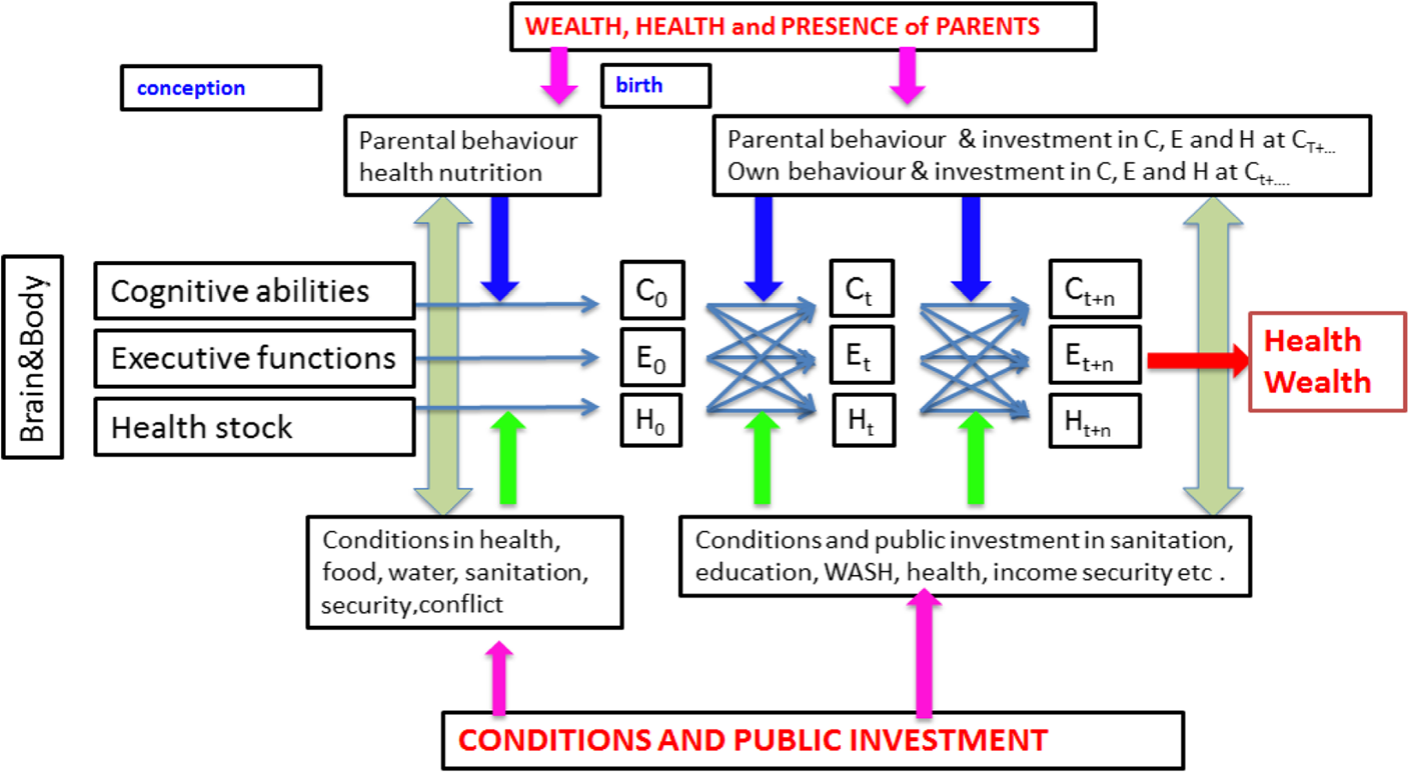
Dynamic life cycle model of the development of wealth and health

In this section we discuss a theoretical framework, which organizes factors mentioned in the previous sections in five big categories: health, executive functions, cognitive development, external circumstances and influence of parents. To account for the role of socioeconomic status of a household in the reproduction of wealth and health over generations and to integrate the findings from neurocognitive science, we propose a dynamic life cycle model that is illustrated in Fig. 1.

The model defines three input variables as a starting point: cognitive abilities (IQ), executive functioning and health stock. These three inputs are essential for the majority of successful life outcomes (e.g. physical health, educational attainment and job market success). The framework tries to explain the development of these input variables into capabilities under the influence of a dynamic interacting environment that includes behaviour and investments from parents and the public environment. The integration of these variables into a dynamic framework explains differences in life outcomes of children and how wealth and health can be reproduced from one generation to the next one.

The framework starts at conception to account for the genetic elements in the definitions of these three input- and environmental variables. However, it acknowledges that between conception and birth, the external conditions and behaviour of the soon-to-be-parents, especially the mother, are critical to the later development of the child. Maternal behaviour (e.g. smoking, alcohol abuse), (mental) health and nutrition have a lifelong effect on a child’s brain development and related cognitive and non-cognitive abilities. Public investments in, for example healthcare, food security, and access to water and sanitation, play a crucial role because they define the situation of the mother. However, the public environment can also have an effect on the unborn. A child for example being born in a conflict area is affected by the stress that environment puts on the pregnant mother.

The dynamic life cycle model follows Heckman (2007) framework in that it explains the development of children and the effect of investments on the formation of capabilities and the processes behind it. These processes relate to the cognitive abilities (C) as well as the non-cognitive abilities (E) (i.e., executive functions) as well as the health stock (H). Produced by the combination of genes and investments and influenced by the environment, the development of capabilities is described as a multistage process (Heckman 2007).

Schematically, between each of the stages (0, t, t + n), behavioural elements of the parents and investments made in the development of a child all influence C, E and H. Later on the child’s owns behaviour and investment play a role as well. Material disadvantage combined with the insecurity and lack of social integration of parents affects the health of those at a progressively lower level of socioeconomic status (Wilkinson and Marmot 2003). Mani et al. (2013) demonstrated that even relatively short spells of poverty have a significant negative impact on the cognitive abilities and executive functions performance of adults. Poor adults are less effective problem solvers and show lower levels of ‘fluid intelligence’. It is very plausible that poor parents have a less effective parenting style because of this reduced performance on their own executive functions and that this parenting style mediates the effects of poverty on their children. Little is yet know about the direct long-term effects of poverty on adults, but it is likely that long-term poor parents are less effective in dealing with their children. Children growing up in poor families therefore suffer from several direct and indirect effects of the parents’ poverty.^4^ Poor mothers are more likely to adopt behaviour that damages the development of the foetus (e.g. smoking during pregnancy) (Wilkinson and Marmot 2003) and are less likely to provide long breastfeeding (Heck et al. 2006). Poor parents have fewer resources available for investment in the human capital of their children (Haveman and Wolfe 1995) and create fewer opportunities for socialising outside the realm of the family (Putnam 2015). These effects are probably combined with a less generous intelligence- and health stock which are both largely genetically coded. All these influence have a direct impact on the health and the performance in the executive functions of the children involved. Health and executive functions interact and (negatively) reinforce each other, as do cognitive abilities and the two other input variables (i.e. health and executive functions). On the other hand, environmental conditions and public investments also have an influence on the development of C, E and H as well.

As convincingly argued by Heckman (2007), the capabilities in C, E and H develop though the process of “self-productivity” where capabilities attained in one stage in life are strengthened by training and cross-fertilization to capabilities in the next stage. This means that if a child is healthier, he/she will also develop more cognitive abilities and his/her executive functions will improve. Similarly, improvement in a child’s cognitive development has a positive effect on his/her health and executive functioning.

Moreover, because capabilities are “dynamic complementary” (Heckman 2007), capabilities produced at one stage in the developmental process raise the productivity of investments in all capabilities at subsequent stages in the life cycle. Therefore, at age‘t’ a child’s status of his/her cognitive abilities, executive functions and health stock is determined by 4 factors (see Fig. 1):

1. The cognitive abilities, executive functioning and health stock a child is born with at *t* = 0;
2. The wealth and health and presence of the parents, which lead to behaviour and financial and cultural investments of parents in the cognitive abilities, executive functions and health stock of the child;
3. The public investments and conditions in education, health, income security and public safety;
4. An interaction among a child’s own cognitive abilities, executive functions and health stock (“self-productivity”).

At age ‘t + n’ that same child’s status of his/her cognitive abilities, executive functions and health stock is being determined by the same factors at age ‘t’ but now there is a cumulative- and interaction effect between abilities (“dynamic complementary”). The behaviour and investments of parents, on the one hand, and public conditions and investments on the other hand, are not seen as completely isolated influences on a child’s development but they interact with each other.

In the end, the framework explains how cumulative investments of parents and the influence of parents’ behaviour conditioned by external developments have an impact on children’s cognitive and health development and his/her executive functioning. Because these effects are “self-productive” and dynamic complementary, parents who “invest” a lot in their children’s development reinforced by a favourable environment will “produce” healthier, more intelligent children with better executive functioning. These effects become larger during the life cycle since the healthier, smarter and better functioning kids will find it easier to learn, will stay healthier and function better than their less fortunate peers. More “investments” in their development will be more fruitful and yield better results through cross-fertilisation between the capabilities and the dynamic complementarity. This illustrates a special version of what could be could called the “Matthew Effect” – Merton (1968). The “Matthew Effect” originated in the sociological field and refers to a verse in the biblical Gospel of Matthew, illustrates the reproduction or even amplification of inequality through intergenerational dependency of educational attainment – in short: the rich get richer, the poor get poorer (Merton 1968).

The previous sections argued that cognitive capabilities (IQ) are only marginally open to positive influences: health and executive functioning are more responsive to investments. “Investments” should not be regarded as financial investment into formal education only. “Investments” in exercising executive functioning are crucial as are investments in physical health. The mechanisms of self-productivity and dynamic complementarity guarantees that individuals with higher level of executive functioning and better health will experience higher returns on investments in formal education.

All in all children whose mothers engage in healthy behaviours during pregnancy, whose early years are spent in a favourable environmental situation, and who experience more investments in C but especially in H and E, will in general function better as adolescents and adults, and will, in turn, become parents who “invest” more in their children. This intergenerational reproduction of wealth and health is produced by a so-called “Droste” effect (recursive). The wealth and health produced by the model at the beginning of adulthood, serves as an input (as parents) for the next generation. The reappearance of the model is recursive because the child’s grandparents form the wealth and health of the parents. Social welfare systems may try to minimize the influence the wealth and health of parents on the life outcomes of their children, but because this parental influence is accumulated over all previous generations, it tends to reproduce itself and to interfere with the influences of public investments.

## Relevance for Framework

The introduction of the dynamic life cycle model reveals both a pessimistic and an optimistic view of the reproduction of wealth and health. The pessimistic view comes from the overwhelming influence from behaviour and investments of parents and their surroundings. Economic studies on the reproduction of inequality through socioeconomic status of the family tell a disappointing story about the fate of the most unprivileged in our societies. However, understanding one of the (major) mechanisms of neurocognitive development (i.e. development of executive functioning), investigating its responsibility for the reproduction of inequality and its dynamic relationships with the other drivers of inequality may provide a more optimistic view.

The separation of cognitive abilities, non-cognitive abilities and executive functions allows for the possibility of social and individual interventions related to the executive functions. Leaving executive functions unpacked together with cognitive and non-cognitive abilities would mask the options that related policy interventions would entail.

### Enhancing Executive Functions

The integrative framework introduced in Fig. 1, including the development of executive functions, gives an innovative perceptive on the battle against the opportunity gap between children growing up in an environment of low wealth and health and an environment of high wealth and health due to the ability to train and enhance executive functions.

An example of an intervention that has been shown to improve executive functioning is a computer-based training. Holmes et al. (2010) have shown that positive effects on working memory after a computer based training with 8 and 11-year-old children were still measureable after 6 months. The study also found a positive effect on math performance 6 months after the training. Another method of training executive functioning is psychical activity. Training consisting of yoga or martial arts (tae kwon do), which focus both on exercise and on character development and mindfulness, has been shown to improve executive functioning (Lakes and Hoyt 2004; Manjunath and Telles 2001).

Diamond (2012) described some general principles that apply to the training of executive functioning. First, children performing the worst on the executive functioning tasks benefit most from the training. Based on this principle and on the link between executive functioning and life outcomes, early childhood training of executive functioning could have great potential to narrow social disparities in academic achievement and health.

A second principle of training is that the transfer effects are limited. This means that children who are trained on a working memory task will show improved performance on untrained working memory tasks, but will show limited improvement on inhibition tasks. However, a training program designed to target multiple components of executive functioning could also have a positive effect on reasoning and general problem solving skills (i.e. higher order executive functions).

As a third principle Diamond (2012) describes, that training should continuously place increasing demands on the executive functioning (i.e. difficulty of the task) of children. To maintain the positive effect of executive function training, the training must follow the increasing development of the child’s abilities and training tasks should be age appropriate. Whether executive functioning improvement can be seen after training also depends on the training time, discipline and practice (Klingberg et al. 2005).

Interventions, which have been shown to improve children’s executive functioning may improve their position in life (Diamond 2012). Interventions aimed to train executive functioning for example have shown to have an effect on academic performance later in life (Holmes et al. 2010). However, more empirical research is required to maximize the effect of these executive functions interventions and their potential role in reducing inequality. This research should on the one hand focus on designing the most effective executive function interventions and on the other hand investigate the presented dynamic life cycle model and the position and role of executive functions within this model.

### Strategies for Empirical Research

Several possible implications for empirical research can be derived from the model. First, the exploration of the intuitive direct links and correlations between the socio-economic status of households, the development of executive functions and their influence on life outcomes can be examined. Tables 1 and 2 give a summary of recent studies providing evidence for those links. However, the full empirical formulation of the theoretical framework needs to go well beyond these direct links to give an accurate view. The dynamic life-cycle model presented simultaneously relates biologically-given starting points, human capital investment (and other) decisions of parents and children, influences of other actors and circumstances, the crucial intermediary role of executive functions, to outcomes in terms of health and wealth (or welfare and wellbeing). Longitudinal studies and birth cohort studies have great potential for answering the research questions related to this model, provided they include an accurate standardized measurement of executive functioning. An indication for this is for example the Dunedin Multidisciplinary health and development study (Moffitt et al. 2011). This study shows that observational ratings of self-control in childhood (i.e. self-reports of impulsive aggression, impulsivity, inattention) predict health, wealth and criminal offending outcomes measured at the age of 32 years. Although the concepts of self-control and executive functions are not interchangeable, they are highly related. Therefore longitudinal research like the Dunedin and other studies form the groundwork for further investigating the dynamic relationship between executive function, cognitive abilities and health.

The obvious challenge for empirical studies along the lines proposed by the framework presented in this paper, is the lack of longitudinal dataset that contain a standardized assessments of executive functioning of respondents at various stages of the life cycle. Obviously the recognition of the potential crucial role of executive functions for explaining differences in life outcomes is a recent innovation brought by neuroscientific studies. Combining, however, standardized measurement of executive functions among children with longitudinal observations on their health, their educational attainments and the socioeconomic status of the parents and changes therein, would allow testing the propositions of the dynamic theoretical framework.

The model formulated in this paper, thus forms the theoretical backbone of a series of empirical studies investigating the determinants of school results and indicators of child wellbeing of boys and girls at the end of primary school, considering (socio-economic) background information on their parent’s household(s), performance in executive functions, individual health indicators and other circumstantial information.

Whether the role of executive functions and their interactions with the other factors and variables in the model have the same strength throughout the full spectrum of the socioeconomic distribution or whether executive functions and the interactions even show the same sign across the distribution, are empirical questions. The answer to these questions is currently unknown: the intended empirical studies expect to contribute to solve at least a part of these puzzles.

In this paper we introduced a dynamic life cycle model that explains the reproduction of wealth and health over generations by merging the perspectives of socioeconomic literature and cognitive neuroscience. The multidimensional construct of cognitive control and monitoring processes is a valuable addition to a framework on reproduction of wealth and health over generations, because executive functions are sensitive to training. Although we know the principles on which an executive functioning training should be based on, the knowledge about how to design an executive functions training that will have a sustainable effect is still under construction (Diamond 2012). Nonetheless the current educational system is already making efforts to enhance executive functioning and the development of a sustainable training program is currently a trending research topic in the cognitive neurosciences.

Given the current developments in the psychological research on the design of effective executive function training interventions and the dynamic lifecycle model introduced in this paper, it is important to clarify the connections and dynamic relationship between executive functioning and other input factors and actors that influences possible life outcome. This could lead to effective policy in (early) childhood education that aims to break the cycle of inequality.

## Footnotes

1 This element is elaborated in the theoretical framework introduced in section 4

2 Healthy life expectancy defined as, the number of years expected to life in good health.

3 That does not fully apply to children in households at the very bottom of the wealth distribution. While their socioeconomic status is difficult to change, increasing income or financial resources may make a significant difference in the life conditions of children. That holds especially in low- and middle-income countries where access to basic social services is neither guaranteed nor free (evidence on this issue is abundant; for a good example see DSD, SASSA and UNICEF. 2012. *The South African Child Support Grant Impact Assessment: Evidence from a survey of children, adolescents and their households*. Pretoria: UNICEF South Africa (Heinrich et al. 2012).

4 Mani et al. (2013) demonstrated that the negative effect of poverty on the performance in executive functions among adult farmers disappeared when the poverty situation was cured. Their study, however, does not allow conclusions on the long-term effects of poverty among adults to be drawn. Equally little known about the damaging effect of poverty on children’s developing brains. For example, the age at which children would suffer directly from poverty in terms of having money-issues is not clear.
